# 3D echocardiography in mitral valve prolapse

**DOI:** 10.3389/fcvm.2022.1050476

**Published:** 2023-01-10

**Authors:** Valentina Mantegazza, Paola Gripari, Gloria Tamborini, Manuela Muratori, Laura Fusini, Sarah Ghulam Ali, Anna Garlaschè, Mauro Pepi

**Affiliations:** ^1^Department of Cardiovascular Imaging, Centro Cardiologico Monzino IRCCS, Milan, Italy; ^2^Department of Clinical Sciences and Community Health, Cardiovascular Section, University of Milan, Milan, Italy; ^3^Department of Electronics, Information and Bioengineering, Politecnico di Milano, Milan, Italy

**Keywords:** mitral valve prolapse (MVP), mitral annulus (MA), mitral regurgitation (MR), three-dimensional echocardiography (3DE), mitral valve surgery, percutaneous mitral valve repair

## Abstract

Mitral valve prolapse (MVP) is the leading cause of mitral valve surgery. Echocardiography is the principal imaging modality used to diagnose MVP, assess the mitral valve morphology and mitral annulus dynamics, and quantify mitral regurgitation. Three-dimensional (3D) echocardiographic (3DE) imaging represents a consistent innovation in cardiovascular ultrasound in the last decades, and it has been implemented in routine clinical practice for the evaluation of mitral valve diseases. The focus of this review is the role and the advantages of 3DE in the comprehensive evaluation of MVP, intraoperative and intraprocedural monitoring.

## 1. Introduction

Three-dimensional (3D) echocardiographic (3DE) imaging represents a considerable innovation in cardiovascular ultrasound in the last decades (1). Advancements in transducer and software technologies allow 3DE acquisition and visualization of cardiac structures from different perspectives (1). Usefulness of 3DE and its superiority over 2D echocardiography has been ascertained in several fields of cardiovascular diseases. Presentation of realistic views of heart valves and their anatomical relationships, accurate quantification of valve geometry, and reproducible volumetric assessment of valves' regurgitation with 3DE color Doppler imaging represent one of the major steps of echocardiographic imaging (1, 2).

In this regard, the assessment of mitral valve (MV) pathology by 3D transthoracic (TTE) and transesophageal echocardiography (TEE) has been incorporated into routine clinical practice, since these imaging methodologies provide the best physiologic and morphologic information on the MV.

This review provides an overview on the role of 3D TTE and 3D TEE in the evaluation of MV prolapse (MVP) and the latest innovations in the assessment of this pathology in terms of anatomic diagnosis, automated and computational analysis of the MV apparatus, eligibility for surgical or percutaneous treatment, pre-operative planning, intraoperative and intraprocedural monitoring.

## 2. Historical background

In the last century, great progress has been made in MVP diagnosis by use of ultrasound. First studies on M-mode echocardiography reported that MVP could be recognized as a mid-systolic posterior and downward movement of echoes from both leaflets toward the left atrium (LA), with a separation of the two leaflets (3). With the advent of 2D probes, MVP was defined as a dislocation of the body of the MV leaflets ≥2 mm above the valvular plane during ventricular systole, as assessed by 2D TTE in parasternal long-axis, or in the apical 3-chamber view (4). Finally, 3DE significantly improved the accuracy in MVP diagnosis by echocardiography and revolutionized qualitative and quantitative assessment of the MV.

3DE finds its roots in the 1970s and early 1980s when efforts were made to develop methods for locating a standard 2D transducer in space, acquiring multiple 2D images, and then melding them into a 3D image (5). After initial experimental techniques (6, 7), rotational transducers and devices became by far the most popular in the early 1990s. However, the breakthrough technology that allowed high-quality real-time imaging was the development, only after the year 2000, of a microbeam former that allowed communication of ~3,000 piezoelectric elements within phased-array transducers (5). Since the initial release of real-time (RT) 3DE in 2002 (8), much research has been performed on this system and a 3D TEE probe was produced in 2006–2007, allowing the initial true clinical application of 3DE.

**Figure 1:**
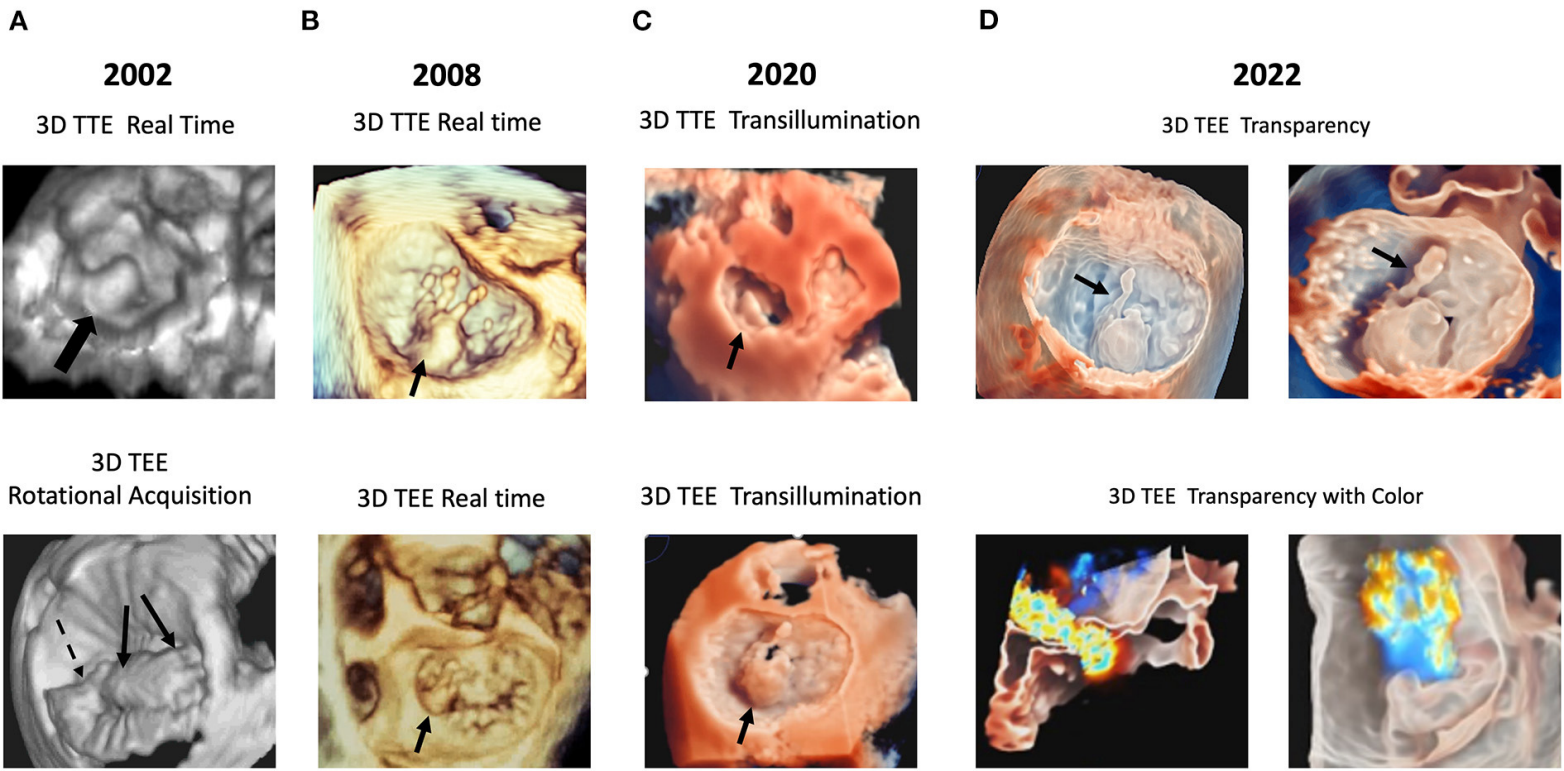
Examples of technical evolution of 3D echocardiography. In **(A)** examples are reported of P2 prolapse by RT 3D TTE (top panel); anterior and posterior leaflet prolapse (arrows) by rotational 3D TEE (bottom panel). In **(B)** examples are reported of P2 flail by RT 3D TTE (top panel), and P1 flail by 3D RT TEE (bottom panel). In **(C)** an example is reported of TI rendering: P2 prolapse by 3D TTE (top panel) and by 3D TEE (bottom panel). In **(D)** an example is reported of the “transparency” effect: P2 flail with a detailed visualization of the ruptured chorda by 3D TTE (top panel); mitral regurgitation color Doppler superimposed to the 3D TEE image (bottom panel). 3D, three-dimensional; RT, real-time. TEE, transesophageal echocardiography; TI, transillumination. TTE, transthoracic echocardiography.

Figure 1 shows examples of 3D TTE and 3D TEE images of the MV with old and new methodologies.

## 3. Clinical applications of 3D echocardiography in the study of the mitral valve

Mitral valve prolapse (MVP) affects about 2–3% of the general population and is the leading cause of MV surgery (9). Prognosis of MVP is mainly determined by the presence and degree of mitral regurgitation (MR). Therefore, identification of morphological and functional factors leading to progression of the MV disease, and finally to surgery, is of utmost importance (10). Analogously, accurate description of the mitral annulus (MA) morphology and leaflets lesions is fundamental to guide the surgeon in MV repair and selection of a proper sized prosthetic ring for annuloplasty (11, 12).

### 3.1. Morphological evaluation of mitral valve leaflets

**Figure 2:**
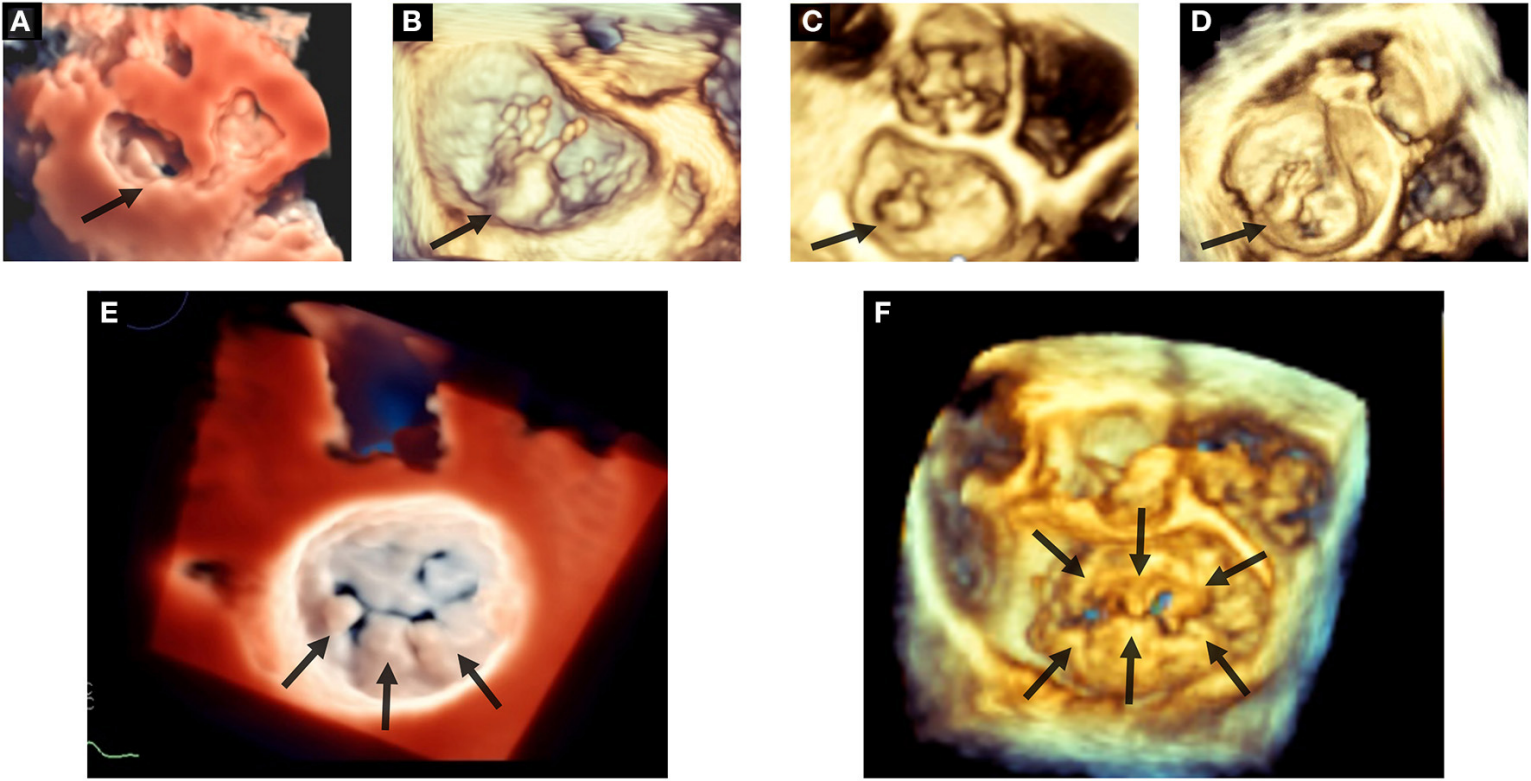
Etiologic phenotypes of mitral valve prolapse. Top panels show examples of fibroelastic deficiency: in **(A)** P2 prolapse by 3D TTE applying the TI effect; in **(B)** P2 flail with multiple ruptured chordae by 3D TEE; in **(C)** P2 flail with single chordal rupture by 3D TEE; in **(D)** P2 flail with multiple chordal ruptures by 3D TEE. Bottom panels show examples of Barlow's disease: in **(E)** prolapse of P1–P2–P3 (arrows) by 3D TEE applying the TI effect; in **(F)** bileaflet prolapse (arrows) by 3D TEE. 3D, three-dimensional; TEE, transesophageal echocardiography; TI, transillumination. TTE, transthoracic echocardiography.

MVP has two main phenotypic expressions: fibroelastic deficiency (FED) and Barlow's disease (BD) (13, 14). FED is secondary to connective tissue deficiency. It usually affects patients >60 years and is characterized by leaflet thickening (due to myxoid infiltration) almost exclusively localized to the prolapsing scallop, and frequent chordal rupture (13, 14). On the contrary, BD is likely genetically determined, and characterized by diffuse myxoid deposition causing excessively thickened leaflets (13, 14). It usually affects younger patients and is characterized by multiple segments involvement, with often bileaflet redundancy, frequently associated with chordal elongation (13, 14) (Figure 2).

2D TTE is fundamental for MVP diagnosis and etiological assessment, MR quantification and hemodynamics estimation. Although in clinical practice the terms prolapse and billowing are used interchangeably, these entities display some differences. For surgeons, prolapse is characterized by a displacement of the leaflet tips and coaptation point above the MA plane, due to chordal or papillary muscle elongation (4, 15). MV billowing is defined when the leaflet body protrudes into the LA above the MA plane, whereas the leaflet tips and coaptation point remain at the level of the MA plane or below (4, 15). Identification of the prolapsing or flail scallops by 2D echocardiographic imaging was improved with the advent of multiplane 2D TEE (16, 17). However, 2D TEE is a semi-invasive technique and still requires the operator to be able to mentally reconstruct the 3D MV anatomy in his/her brain based on multiple acquisitions of 2D images (9, 18).

3DE aids in differential diagnosis between MV prolapse and billowing, and between organic MV lesions and other potential etiologic causes of MV flail, such as infective endocarditis causing leaflet flail/perforation or post-ischemic leaflet flail secondary to complete or partial rupture of papillary muscle. Additionally, 3DE aids in the identification of the prolapsing or flail scallops.

Different methods for 3DE data acquisition are available: RT 3DE (simultaneous multiplane mode, and real-time 3DE) and ECG-triggered multi-beat 3DE (zoom and full volume mode) (1). RT 3DE consists in the acquisition of a volume data set in a single heartbeat and is limited by the low volume rate in case of large volume data sets (19). This issue may be overcome by multi-beat acquisitions during single breath-hold, with subsequent post-processing of the volume data set and 3D rendering (19). The zoomed acquisition provides the highest temporal and spatial resolution and is the preferred mode of acquisition to evaluate leaflet anatomy and function in detail. On the contrary, full-volume acquisitions are mainly applied when the operator needs to investigate the entire MV apparatus (17).

Differentiation between prolapse and billowing by conventional 2D echocardiography is not always straightforward, due to the non-planar leaflet-annulus relationship (2). Addetia et al. showed that the use of 3D color-coded parametric models of maximal leaflet displacement from the MA plane provides detailed information to differentiate leaflet prolapse from billowing and increases the accuracy in the identification of the site and extent of MV lesions, also by operators with limited experience (15). By providing the “en-face” view of the valve from the atrial perspective (the so-called “surgical view”), 3DE also allows a comprehensive evaluation of the MV morphology and a simultaneous visualization of all mitral scallops (20–23).

With the introduction of 3D matrix transducers, it became possible to identify the prolapsing or flail segment by using multiple 2D x-plane views acquired from a standard parasternal short axis (24). The cursor in a primary image is placed in correspondence of a region of interest, then an orthogonal view of the same region is simultaneously displayed as a secondary image resembling a parasternal long axis view (24). By making a medial-to-lateral sweep across the MV coaptation line in the short axis view, a segmental analysis of the entire MV from the posteromedial to the anterolateral commissure can be performed, allowing the detection of the prolapsing or flail segment in the secondary image (24). The limit of the x-plane technique is the lower frame rate in contrast with the original 2D image (24).

In early 2000's, with the advent of new transthoracic 3D matrix array probes that allowed RT 3DE rendering, 3DE proved to be accurate and reproducible in localizing the prolapsing segment with a higher accuracy than corresponding 2D imaging, assuming surgical findings as reference (25). Diagnostic accuracy of 3D TTE vs. 2D TTE was even more evident in complex prolapse, with involvement of the anterior leaflet, both leaflets, and/or commissures (25, 26).

With subsequent advancements in matrix technology that allowed to perform 2DE, 3DE and color Doppler mode with a single TT transducer, another study including 149 patients with MVP, confirmed a high accuracy of 3D TTE in the localization of the prolapsing or flail scallop, as compared to surgical inspection (sensitivity 89%, specificity 94%, overall accuracy 93%) (27). Accuracy was slightly but significantly lower with 2D TEE (sensitivity 84%, specificity 94%, accuracy 91%), which was mainly due to the lower sensitivity of 2D TEE in the detection of lesions located in the posteromedial commissure (27).

In 2014 Ben Zekry et al. demonstrated that all four imaging modalities (2D TTE, 2D TEE, RT 3D TTE, and 3D TEE) were comparable in identifying MR etiology, but 3D TEE had the best agreement with surgery in the identification of anterior leaflet prolapse and bileaflet or multisegmental prolapse (16). Indeed, 3D TEE allows a more authentic depiction of MV morphology, providing a prompt identification of individual scallops and characterization of morphologic leaflet variants and commissures (28, 29). Biaggi et al. found that 3D TEE was more accurate than 2D TEE in specific conditions, such as morphological evaluation of complex MVP and quantitative evaluation of MV leaflets and annulus, assuming surgical valve inspection and measurements as reference (30). Additionally, in less experienced echocardiographic readers, 3D TEE allows a significantly shorter time to MVP diagnosis, higher diagnostic accuracy (mainly due to higher specificity), and improved identification of P1 and P3 prolapse compared to 2D TTE and 2D TEE (9).

Consequently, since 2000's, RT 3D TTE has been suggested to be integrated in routine echocardiographic evaluation of the MV disease and become part of everyday clinical practice, performing 2D and 3D TEE examination intra-operatively to refine the diagnosis or pre-operatively in dubious cases (25–27, 31). Moreover, 3D TEE has become the reference imaging modality to guide percutaneous MV procedures.

**Figure 3:**
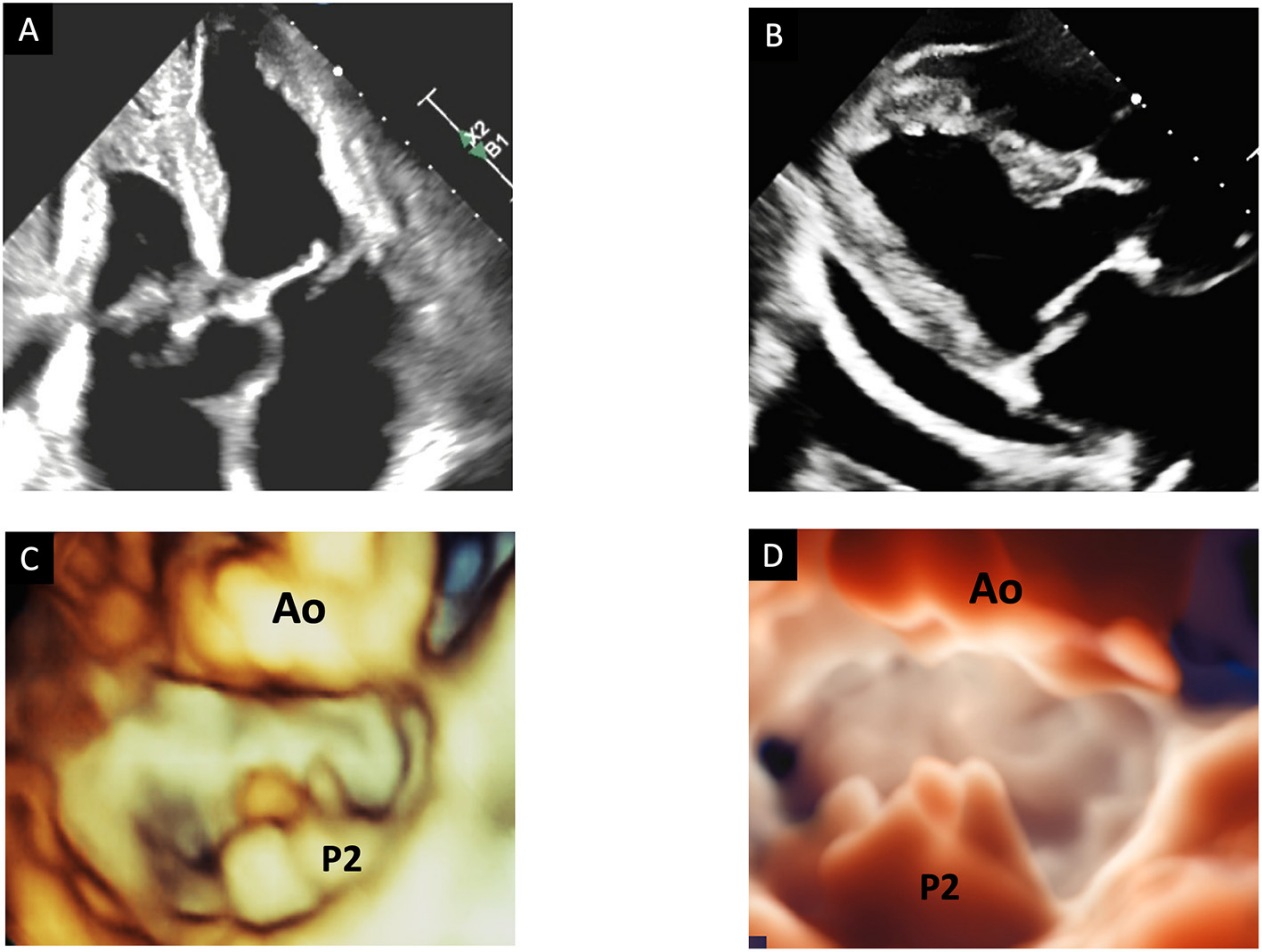
The advantage of transillumination rendering in 3DE. Top panels show an example of P2 flail by 2D TTE with evident eversion of the scallop in a 4-chamber view **(A)** and in a long-axis view **(B)**. Bottom panels show 3D TTE surgical views of the mitral valve by standard 3D reconstruction **(C)** and by TI rendering **(D)**. Only TI clearly shows the entire P2 scallop prolapsing in the left atrium with multiple chordal ruptures. 3D, three-dimensional; TEE, transesophageal echocardiography; TI, transillumination. TTE, transthoracic echocardiography.

**Figure 4:**
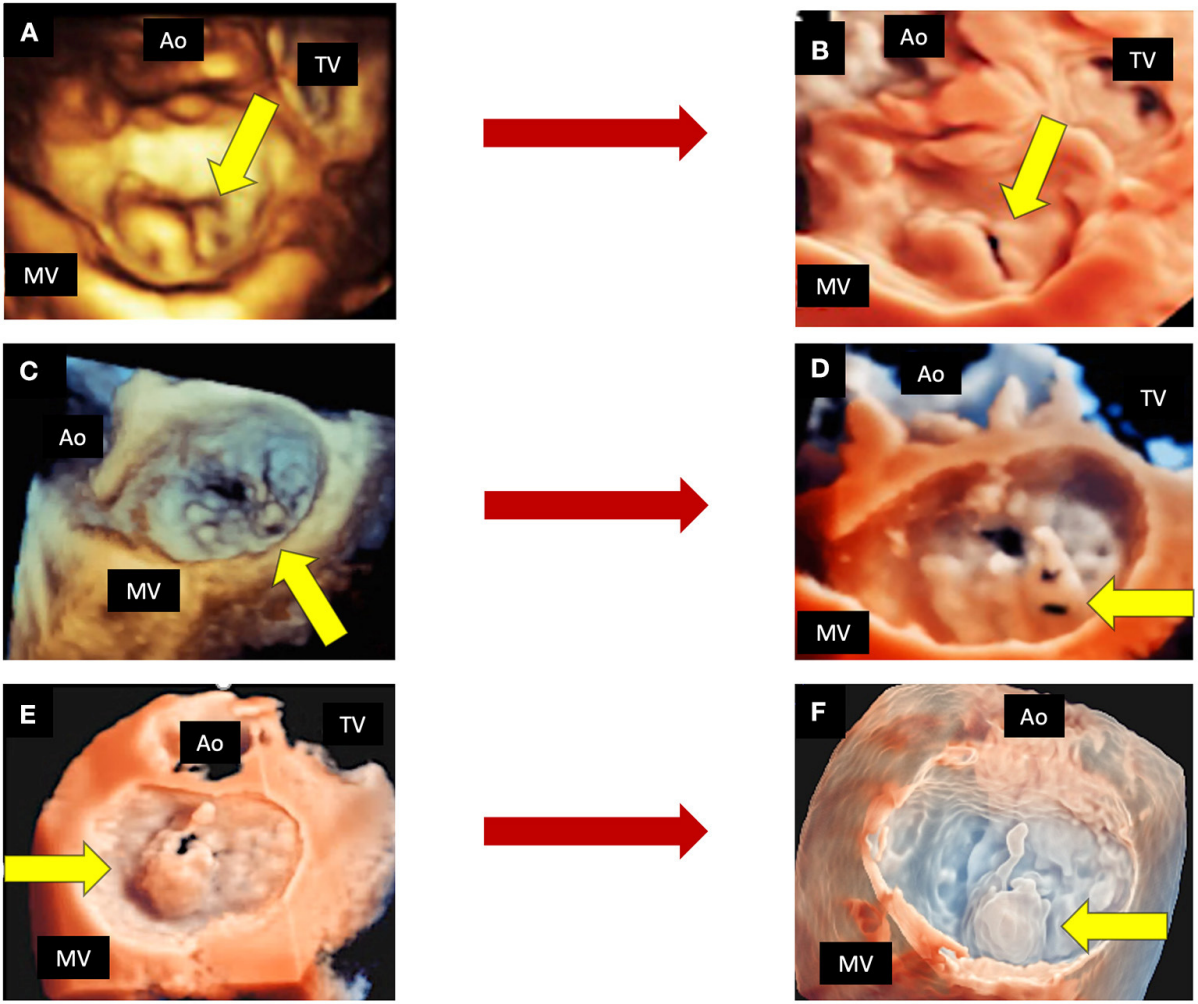
The role of new 3D tools in echocardiography. Shown are three examples of MVP, in which new tools may improve the quality of imaging. Top panels show a P2 prolapse by standard 3D TTE **(A)** and by TI rendering **(B)**. Mid panels show a complex P2 prolapse by standard 3D TEE **(C)** and by TI rendering, which clearly shows the fragile texture of the leaflet **(D)**. Bottom panels show a P2 flail by 3D TEE applying the TI tool **(E)** and the transparency effect **(F)**. 3D, three-dimensional; Ao, aorta; MV, mitral valve; TEE, transesophageal echocardiography; TI, transillumination. TTE, transthoracic echocardiography; TV, tricuspid valve.

More recently, new 3DE tools have been introduced into clinical practice, that improve the visualization of cardiac structures. Transillumination (TI) introduces shadow effects by using a virtual light source into the data set (32, 33). In a small series of MVP patients undergoing surgery for significant MR, assuming surgical findings as the gold-standard, it was shown that compared to standard 3DE, TI showed significantly higher accuracy in recognizing prolapsing scallops, and chordal rupture (32). Transparency or “glass” effect allows the operator to adjust tissue transparency, thus improving the delineation of cardiac and extracardiac structures (34). This tool showed an incremental value compared to TI and standard 3D technology in the recognition of MV anatomy, border delineation, and pathogenetic mechanisms (35) (Figures 3, 4).

#### 3.1.1. Cleft mitral valve

MV cleft is a congenital indentation of the MV. Since the posterior leaflet has physiologic indentations, diagnosis of posterior leaflet cleft was proposed when the indentation depth is > 50% of the depth of the adjacent leaflet tissue or when it reaches the MA. In MVP, localization, size and shape of clefts and posterior leaflet commissures are fundamental to guide surgical and interventional procedures in case of significant MR (36, 37). Direct suture or insertion of an autologous pericardial patch, with or without annuloplasty, are the most effective surgical techniques to treat MR secondary to MV indentations (36, 37). Though clefts represent an unfavorable anatomical feature for MitraClip implantation, preliminary data suggest that it might be an option to correct MV indentations in selected patients at high surgical risk (37–39).

By 2D TTE, the parasternal short-axis view at the level of the MV is the optimal view to detect leaflet indentations, that appear as an area of discontinuity of MV echo at end-diastole (36). However, indentations diagnosis by 2D TTE is particularly challenging, and it is rather difficult to identify their shape and maximum size, possibly because of echo dropout, artifacts and because the leaflets and the MA are placed at different levels (36, 37).

RT 3DE displays indentations in multiple views, allowing direct identification of their position, shape, and size (36). Assuming surgical findings as reference, 3DE has a significantly higher diagnostic accuracy compared with 2D echocardiography (36). Similarly, Narang et al. investigated by 3D TEE the prevalence and locations of isolated cleft MV in patients with unexplained ≥ moderate MR (37). They found that isolated cleft prevalence was significantly higher (3.3%) than previously reported on the basis of 2D echocardiographic studies (37). By using 3D color Doppler, the origin of the regurgitant jet can confirm the cleft position. Therefore, 3DE is a valuable tool to detect mechanisms of unexplained significant MR, and TI recently proved to have a significantly higher accuracy than standard 3DE in recognizing leaflet indentations (32).

### 3.2. Evaluation of geometry and mechanics of the mitral valve annulus

The MA is a complex asymmetrical 3D saddle-shaped fibrous structure, separating the LA from the LV (40). Anteriorly, it is connected to the aortic annulus by the mitro-aortic curtain; the posterior annulus is composed of a discontinuous connective string interspersed with adipose tissue, connecting the LA wall to the LV myocardium and sustaining the posterior mitral leaflet (41–43). The “saddle” of the MA is characterized by two peaks located anteriorly and posteriorly, respectively, whereas the lowest points are located medially and laterally at the commissures (41, 42). This peculiar geometry of the MA, in conjunction with leaflet billowing, allows the unloading of the MV leaflets from excessive systolic forces, ensures leaflet coaptation, and promotes LA and LV filling and emptying (41, 42, 44).

The MA is a dynamic structure, showing three types of motion throughout the cardiac cycle. The *sphincteric contraction* is determined by the passive movement of the posterior MA, following contraction and relaxation of the LV basal wall (41, 43). This movement corresponds to a reduction in the antero-posterior (AP) diameter and allows coaptation of the leaflets before the LV pressure increases (41, 43). The *translation motion* derives from the reduction of the LV long axis due to the contraction of myocardial fibers (41, 43). It corresponds to the approximation of the MA toward the LV apex (41, 43). The annular *folding* across the intercommissural (IC) diameter corresponds to an accentuation of the MA saddle-shaped geometry, which furtherly reduces the systolic stress exerted on the leaflets (41, 43). Normal MA dynamics is characterized by early systolic area contraction and accentuation of the saddle-shape due to AP diameter contraction, increase in leaflet tenting height and apical descent of commissures (13, 45–48). After leaflet closure, the AP diameter expands, leaflet tenting height decreases and the MV area gradually increases until end-systole (13, 45–48).

Quantitative evaluation of the MA enlargement and dysfunction, together with morphological evaluation of the MV leaflets, aids in the understanding of MR pathophysiology and is essential in the planning of surgical and percutaneous MV repair (13, 45, 48).

**Figure 5:**
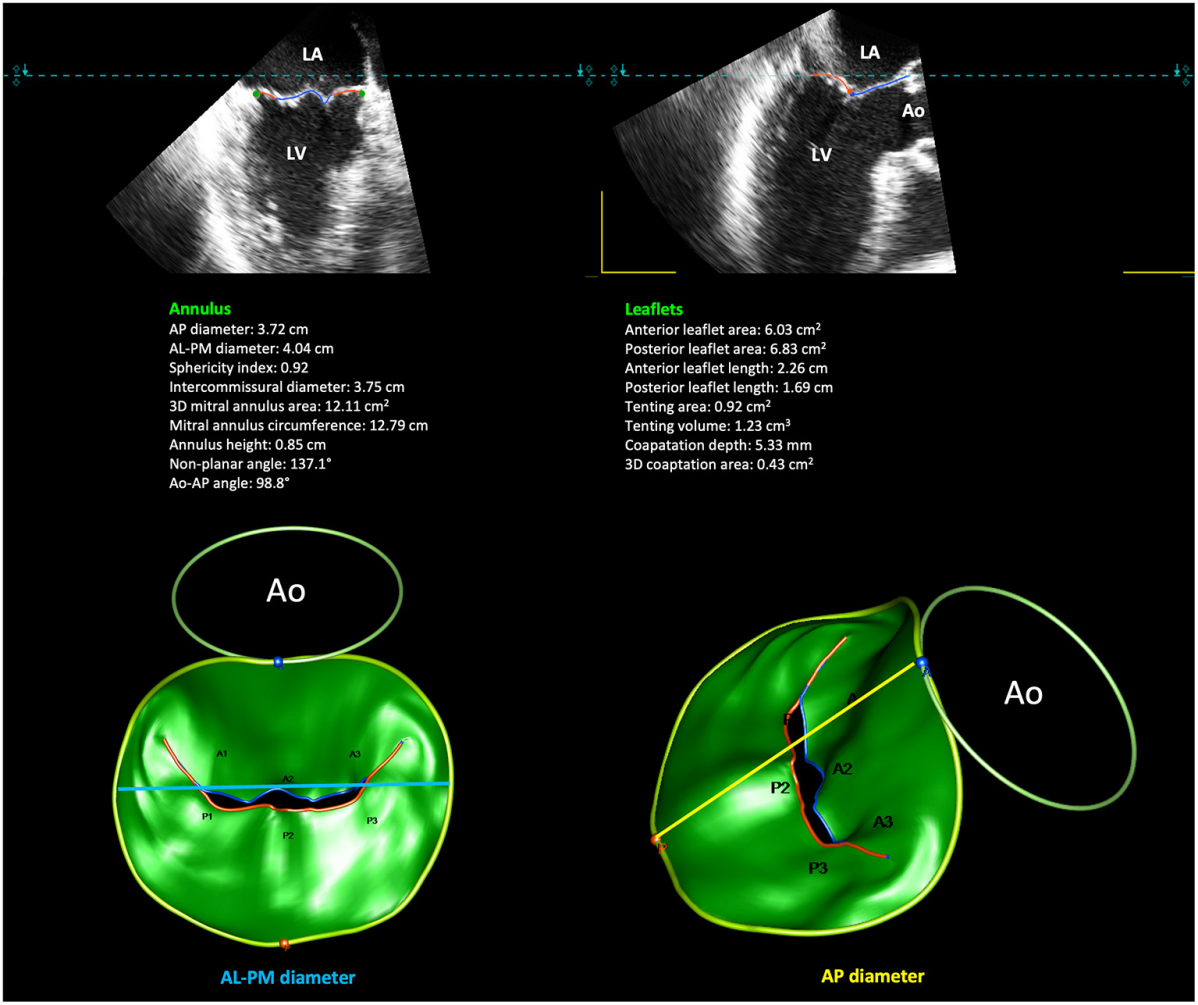
Quantitative modeling of the MV leaflets and annulus by 3DE. Shown is an example of 3D MA reconstruction and semi-automatic computation of 3D MA and leaflets measurements in a case of P2 prolapse. 3DE, three-dimensional echocardiography. MA, mitral annulus.

2D evaluation of the non-planar MA structure is necessarily incomplete and is limited to its visual anatomical evaluation, and to linear or area measurements (48, 49). MA quantification by 2D TTE includes measurement in diastole of the AP diameter (in the parasternal or in the apical long-axis view), and septo-lateral diameter (in the apical 4-chamber view) (11, 23). Using the parasternal long-axis view, annular dilatation is defined by an annulus/anterior leaflet ratio >1.3 o by an AP diameter >35 mm (23). However, MA diameters tend to be underestimated by 2D echocardiography compared to RT 3DE (11). Several commercially available 3DE software systems provide a detailed high-resolution static and dynamic reconstruction of the MA by tracking landmarks throughout the cardiac cycle, allowing the identification of any structural annular deformation and abnormalities in leaflets morphology (10, 12, 46, 48, 50). The automatic tracking workflow finally delivers a 3D rendering of the MV and provide 3D parameters of the MA and leaflets: AP diameter; anterolateral-posteromedial (ALPM) diameter; IC diameter; MA height; MA circumference and 3D area; sphericity index; aortic-to-mitral plane angle; planarity index, intended as a surrogate of the annular saddle-shape; non-planar angle; MV 3D tenting height, area, and volume; leaflets length and 3D area; length, height and area of coaptation (10, 12, 46, 48) (Figure 5).

Compared to healthy subjects, patients with MVP show significantly larger MA size, being both AP and ALPM diameters longer and 3D MA area larger than controls (10, 12, 46, 47, 51), with BD showing greater MA dimensions, higher mitral height, and increased leaflets area than FED (12, 13). Additionally, MVP is characterized by increased ellipticity, reduced height and flattening of the MA compared with controls, as well as increased length and surface area of the leaflets, higher billow height and volume, and longer coaptation length (10, 42). Lee and colleagues demonstrated that structural deformation of the MA and MV leaflets were associated with higher degrees of MR. Specifically, predictors of significant MR in MVP were increased annular size and flattening, leaflet billow volume and height, and coaptation line length (10). They argued that annular flattening would increase the stresses exerted on the leaflets and favor the progression of leaflet lesions, including chordal rupture (10).

Several studies, using 3D TTE or 3D TEE, have also been conducted to analyze the annular dynamics in MVP, which is quite disputed (13). Some authors reported a preserved MA function in MVP patients (46, 47, 51). Conversely, other studies observed a significant decrease in early-systolic MA area contraction, due to reduced AP diameter contraction and simultaneous IC diameter expansion, as well as a delayed and attenuated saddle shape accentuation, and late-systolic abnormal increase in MA area (13, 19, 46). When considering FED and BD as separate entities, MA function and dynamics in FED are close to normal, whereas MA is excessively enlarged, flattened and dysfunctional in BD. Indeed, BD show a significant decrease in early-systolic AP contraction and a late-systolic increase in IC diameter (13, 52). These features might influence the choice of repair technique and the selection of annuloplasty ring (52).

Thanks to the progressive improvement of 3D scanners, the evaluation of the MA by TTE has become feasible, accurate, and reproducible. In 2014, Mihaila et al. showed that 3D TTE results were similar to those obtained by 3D TEE. Quantitative evaluation of the MA is compromised mainly in case of suboptimal apical acoustic window, major calcification of the MA, and irregular heart rhythm (48). Additionally, the advent of new software providing full-automatic measurements of 3D echocardiographic static and dynamic characteristics of the MA, requiring minor or no manual adjustments, has rendered the analysis less time-consuming and more reproducible, with a significant impact on clinical practice (53).

#### 3.2.1. Mitral annular calcification

Mitral annular calcification (MAC) is a degenerative/inflammatory process resulting from progressive calcification of the posterior annulus, and affecting up to 15% of population >70 years or patients with multiple cardiovascular risk factors or with chronic kidney disease (43, 54). The posterior MA is not supported by a rigid connective structure and may undergo microinjuries at the junction between the leaflet and the LV wall (43). The process can be accelerated by pathologic conditions increasing the hemodynamic stress over the posterior MA, such as systemic hypertension, aortic stenosis, hypertrophic cardiomyopathies, or MV prolapse (43). Identification of this relatively benign lesion is essential before surgery (MV repair or replacement) and before percutaneous procedures, as it affects the success of interventions on the MV.

By 2D echocardiography MAC appears as a bright, echodense mass in the region of the posterior MA, with a crescentic shape in the parasternal short-axis view (55). Caseous calcification appears as a highly echogenic mass, with an echolucent core, indicative of liquefaction (55). Differential diagnosis between MAC, thrombus, tumor, and vegetation by 2D TTE can be sometimes challenging (56).

In 2010, Assudani et al. presented a case in whom 3D TEE allowed a more confident diagnosis of caseous MAC, compared with 2D echocardiography and 3D TTE. 3D TEE revealed an echodense mass involving the posterior MA, and a relatively less echogenic area characterized by multiple, small echodensities surrounded by highly echogenic borders, consistent with regions of calcific granules interspersed in a liquefied substance (55). Such echodensities could not be detected by 2D echocardiography, mostly due to shadowing and reverberations caused by calcium (55). Recently, a case report was published where a 3D “en-face” MV view by TEE was crucial to understand the nature of MAC (56). The application of TI technology allowed the distinction of MAC from surrounding structures, since calcific areas cannot transmit the light generated by the virtual light source of TI (56).

#### 3.2.2. Mitral annular disjunction

A recently described annular anatomic abnormality is the MA disjunction (MAD), which is described as a separation between the LV myocardium and the attachment of the posterior MV leaflet to the LA wall (57). MAD has been variably described as a simple anatomical feature of the MA, a potential cause for myxomatous MV degeneration, or as a risk factor for ventricular arrhythmias (58–64). MAD can be assessed by different imaging techniques (TTE, TEE, cardiac computed tomography, and cardiac magnetic resonance) (59–62, 64–66). Regardless of the imaging modality used, MAD is evaluated at end-systole, and is measured as the distance between the posterior leaflet hinge point and the adjacent LV basal wall. To detect MAD and measure its maximal length by echocardiography, a frame-by-frame analysis of high-resolution and excellent-quality images is required.

By 2D TTE and TEE, MAD is evaluated in standard long-axis views, so that the entire MA circumference cannot be completely assessed for MAD presence (58, 67). However, histological studies showed that MAD can be found in any segment of the MA, with the exception on the mitro-aortic curtain (63). Indeed, the posterior two-thirds of the MA may represent an area weakened by mechanical stress (68).

3D echocardiographic imaging can be used to comprehensively evaluate the entire MA. Lee et al. analyzed the MA anatomy and dynamics by 3D TEE, using multiple reconstructed planes at 10° intervals around the long axis. Comparing MVP patients with MAD with those without MAD, and with controls, they found that MAD is associated with paradoxical dilatation and flattening of the annulus in systole, as well as accentuated leaflet abnormalities (62). In the presence of a disjunctive annulus, the annulo-ventricular coupling is lost and the MA follows the atrial wall motion (60, 62, 68), undergoing a systolic expansion and flattening, which is supposed to increase the mechanical stress exerted on mitral leaflets (67). Such abnormalities would lead to an acceleration of the degenerative process and a progression of the underlying MV disease (58, 62, 67). Therefore, evaluation of MAD presence has become integral part of standard TTE and TEE when assessing patients with MVP.

### 3.3. Quantification of mitral valve regurgitation

In MVP patients, prognosis, and timing for surgical or percutaneous treatment depend on MR severity, which needs to be accurately quantified (17, 69). 2D TTE is the first-line non-invasive examination performed for quantification of MR severity, its hemodynamic and ventricular consequences (70). Technological improvements have made 3DE more accurate and reproducible in quantifying the MR grade, than 2D echocardiography imaging, which has several limitations and necessitates a multiparametric approach for MR quantification (17, 69, 71).

Assessment of MR severity by 2D echocardiography relies on qualitative, semi-quantitative, and quantitative methods. Qualitative methods include the visual assessment of the color wave (CW) Doppler intensity of the MR jet (69). Semi-quantitative methods include color flow Doppler imaging, the vena contracta (VC) width, the antegrade velocity of the mitral inflow, and the pulmonary venous flow pattern. The VC is the narrowest width of the regurgitant jet occurring at or immediately downstream of the regurgitant orifice (69). However, it assumes that the regurgitant jet origin is circular. Moreover, VC width measurements by 2D echocardiography are not additive in case of multiple jets, and intermediate values need confirmation with other methods (69). Among quantitative methods, the proximal isovelocity surface area (PISA) method for the calculation of effective regurgitant orifice area (EROA) and the regurgitant volume is the most used and valuable to quantify MR, whenever feasible (69). However, it is influenced by systolic changes in regurgitant flow, and it is just an instantaneous measure of a single-frame peak flow rate (72). This limitation may be more relevant in case of mid- or end-systolic than holosystolic MR. Furthermore, the PISA method is less accurate for eccentric jets and for jets with non-circular regurgitant orifice (72). Finally, this method is not validated for multiple jets, and errors in the application of this method are finally squared.

3D color Doppler acquisition adds flow information onto 3DE anatomy (1). It is acquired using live 3D or multi-beat full-volume acquisitions, being the first more limited by lower frame rates, and the latter more affected by stitching artifacts (1). For detailed color flow analysis, 3D TEE is recommended over 3D TTE because it provides color Doppler images of better quality (1). The 2D PISA method relies on the assumption that the isovelocity shell is hemispheric (73). However, this may not hold true for eccentric or multiple jets, as well as for non-circular regurgitant orifices (69). Direct evaluation of the VC area (VCA) by 3DE showed significant asymmetries of the regurgitant orifice, questioning the assumption that PISA has a spherical morphology and VC a circumferential shape, thus suggesting that single-plane measurements might be inaccurate (71, 74). From 3D color Doppler data set, 3DE allows direct measurement of 3D VCA, without any flow or geometric assumption (71, 74). After cutting the 3D volumetric data set to optimize the visualization of the regurgitant jet from the “en-face” view, alignment of planes is manually adjusted to identify the origin of the jet (75). Specifically, two orthogonal planes (*x* and *y*) are oriented along the major axis of the regurgitating jet, with the third plane (z) placed perpendicular to the direction of the jet and passing through the cross-sectional area of the VC (75). 3D VCA is then measured as a planimetry of the regurgitant orifice area (17). This method is proved to be more reliable and accurate than the 2D VC method for MR quantification due to direct visualization of the orifice morphology (76, 77). Assuming the 2D integrative method as the reference standard for MR grading, a cut-off value of 0.41 cm^2^ for 3D VCA showed 82% sensitivity and 97% specificity in differentiating moderate from severe MR (78). Using 3D VCA and sampling the regurgitant jet with the CW Doppler, the regurgitant volume can be calculated multiplying the anatomical regurgitant orifice area and the velocity time integral, similarly to what is done with 2D echocardiography. Shanks and coauthors found that 2D TEE underestimated EROA and the regurgitant volume compared with 3D TEE and cardiac magnetic resonance, and the underestimation of the regurgitant volume was significantly more evident in eccentric than central MR (79). In case of multiple jets, the sum of 3D VCAs accurately reflect MR severity (77). Another way to assess MR grade by 3DE is using the 3D PISA technique or direct measurement of the anatomic regurgitant orifice area (AROA) (74). The first consists in measurement of the 3D surface of the proximal flow convergence region without any geometric assumption (74). Spampinato et al. prospectively evaluated the diagnostic accuracy of the 3D PISA method in comparison to standard 2D echocardiography, and cardiac magnetic resonance, using a multiparametric 2D TTE approach as reference for assessing the MR grade in a population of MVP patients (80). They showed that semi-automated RT 3DE derived PISA may allow similar MR grading as compared to standard 2D TTE, with overestimation in case of asymmetric flow convergence zone or when assuming cardiac magnetic resonance as reference (80). The authors argued that 3D PISA has the advantage to display the localization and extension of the proximal flow convergence region from the “en-face” view, aiding the observer in the evaluation of MVP complexity (80). The AROA is a direct measure of the regurgitant orifice, rather than a measure derived from hemodynamic data (81). Its calculation requires a multiplanar reformatting process of 3D data set: using 2D cut planes, a third plane is placed at the gap between the prolapsing or flail leaflet, then the orifice area is traced (74, 81).

A limit of 3DE for MR quantification is the lower temporal and spatial resolution of 3D color Doppler imaging, due to intrinsically lower frame rates, as compared to 2DE (71). This may constitute a problem especially in the presence of small regurgitant orifice area (74). Second, 3D VCA, 3D PISA and 3D AROA measurements require adequate expertise and are time-consuming since they are based on off-line post-processing. Third, there is no standardization of the temporal frame, where measurements should be performed (17). The choice of the systolic frame affects VCA assessment and may cause interobserver variability and low reproducibility (74). In case of late-systolic MR, alike 2D, 3D measurements are performed at the time-point corresponding to maximal MR grade, which may not reflect the entire systole, and so overestimate MR severity. Finally, 3D VCA and 3D AROA depend on multiplanar reconstruction to obtain the cross-sectional plane of the regurgitant orifice (74). Particularly in case of eccentric jet, a non-perpendicular cropping of the regurgitant jet may cause an overestimation of VCA (74). Despite several studies have documented the higher accuracy of 3D vs. 2D assessment of MR degree, pitfalls of 3D calculations limit 3DE application in routine clinical practice for MR assessment (17).

Table 1 summarizes the incremental role of different echocardiographic modalities in the quantitative and qualitative evaluation of the MV.

**Table 1:** Incremental role of 3D echocardiography in the evaluation of mitral valve prolapse.

	**2D TTE**	**2D TEE**	**Standard 3D TTE**	**Standard 3D TEE**	**Advanced 3D TTE**	**Advanced 3D TEE**
Feasibility	+++	++++	+++	++++	+++	++++
Accuracy in the evaluation of MV morphology	++	+++	+++	++++	+++	++++
Differentiation between simple and complex MV prolapse	++	+++	+++	++++	+++	++++
Characterization of MV leaflets texture	+	++	++	+++	++	++++
Chordal evaluation	++	+++	+++	+++	+++	++++
Cleft detection	+	++	++	+++	+++	++++
MA measurements	+	+	++	+++	++	+++
MR quantification	++++	++++	+++	+++	+++	+++
Accuracy in the identification of MR origin	++	+++	+++	+++	+++	++++

2D, two-dimensional; 3D, three-dimensional; MA, mitral annulus; MR, mitral regurgitation; MV, mitral valve; TTE, transthoracic echocardiography; TEE, transesophageal echocardiography. The role of 3DE in the evaluation of MVP is graded in symbols, in a scale from + to ++++.

## 4. Surgical treatment of mitral valve prolapse

Surgical MV repair is the reference standard treatment for severe degenerative MR, since it is associated with better survival compared to MV replacement. The recent guidelines from ESC and EACTS recommends surgery in patients with symptomatic severe primary MR and acceptable surgical risk according to the Heart Team (82). The presence of LVEF ≤ 60%, LVESD ≥40 mm, LA volume ≥60 mL/m^2^ or diameter ≥55 mm, systolic pulmonary arterial pressure >50 mmHg and atrial fibrillation, are considered triggers for intervention regardless of symptomatic status (82). Moreover, guidelines underline that, even in asymptomatic patients with preserved LV systolic function, MV repair should be considered when the likelihood of a successful repair is high and in presence of a low operative risk (82). Surgical outcomes depend on pre-operative status, mechanism of MR, MV anatomy, technique of repair, and experience of the center and surgeon. Centers with large experience in MV repair achieve hospital mortality <1%, very low rates of major adverse events and good long-term results (83–88). Long-term survival and quality of life after timely MV repair mirror the age-matched general population (89). In contrast, late survival is reduced if MV repair is carried out in patients with congestive heart failure, reduced LV ejection fraction, pulmonary hypertension, or atrial fibrillation (89).

Characterization of MV anatomy is fundamental to plan the most appropriate repair technique, and to predict the surgical outcome (9, 18, 27, 30, 31, 90, 91). Table 2 summarizes parameters that should be assessed by 3DE before surgical MV repair.

**Table 2:** Pre-operative checklist of 3D echocardiographic parameters for planning MVP surgery.

**Qualitative parameters**	
Number of involved scallops	
Commissural or anterior leaflet involvement	
Leaflet abnormalities (clefts or calcification)	
Annular calcification	
Identification of multiple regurgitant jets	
**Quantitative parameters**	**Normal values**
3D VCA (cm^2^)	≤ 0.4 (cut-off for severe MR)
MA circumference (mm^2^)	106 ± 10
MA area (mm^2^)	738 ± 125
P2 height (mm)	≤ 20
A2 height (mm)	≤ 26
Intertrigonal distance (mm)	30 ± 3

3D, three-dimensional; MA, mitral annulus; MR, mitral regurgitation; VCA, vena contracta area. Modified from Pastore et al. (75).

### 4.1. Surgical mitral valve repair

The main goals of MV repair (restitution of physiological leaflet motion, achievement of adequate leaflet coaptation and annular stabilization with maintenance of an adequate mitral orifice) can be achieved using a variety of isolated or combined techniques (leaflet resection, implantation of artificial chordae, chordal transposition/transfer, edge-to-edge technique, annuloplasty using a prosthetic ring or band) according to the type and location of the mitral lesions. Currently, >95% of degenerative MV lesions can be successfully repaired in experienced centers. The valve abnormality that lends itself to a successful repair is the degenerative disease with FED or chordal rupture involving a scallop of the posterior leaflet (92). In patients with this condition, quadrangular or triangular resection with implantation of an annular ring will result in successful repair. Repair becomes more difficult in the presence of MAC, precluding adequate ring annuloplasty, or calcification and fibrosis of the valve leaflets. Increasing leaflet redundancy and excessive tissue involving the anterior leaflet need a higher level of surgical expertise and additional surgical procedures such as chordal transfer and implantation of artificial chords, as well as addressing commissural problems (88, 93–97).

Since more complex surgical procedures are mainly indicated in cases with more complex prolapses and vice versa, noninvasive pre-operative assessment of the MV anatomy is essential to define feasibility and complexity of repair. 3DE, eventually using ad *hoc* post-processing software, may facilitate surgical planning, applying a tailored approach to each patient (2, 16, 98). The relationship between the extent of MV lesions, as assessed by RT3DE, and the complexity of surgical procedures has been demonstrated in the literature (31). Involvement of the anterior leaflet, bileaflet and multisegmented prolapse, severe annular calcification, increased annular dimensions and leaflet height were shown to be anatomic predictors of a lower likelihood of successful repair (30). Besides the ability to predict the complexity of surgical repair, 3DE has been demonstrated to predict residual MR and cardiac reverse remodeling at long-term follow-up (91). Indeed, Tamborini et al. found that complex prolapses, undergoing complex procedures, had twice the percentage of residual MR ≥2 after MV surgical repair vs. simple MV lesions undergoing simple procedures. Favorable cardiac remodeling, observed in all cases at 6-months follow-up, was maintained at 3 years only when MR was <2 (91). A machine learning-based prognostic model was developed and tested to predict the risk of MV repair failure and MR recurrence based on pre-operative clinical, 2D and 3D TTE data (99). Patients with a complex prolapse, specifically with A2 prolapse, more frequently underwent MV replacement or showed early failure of MV repair. These findings suggest that, in the future, machine learning could have an important clinical role in evaluating prognosis in patients undergoing MV repair for MVP (99).

### 4.2. Surgical annuloplasty

Several publications have underlined the importance of MV annuloplasty during repair of degenerative MV disease to optimize repair durability (100, 101). Debate persists regarding the superiority of partial band vs. complete ring devices. Suri et al., using RT 3D TEE, demonstrated the presence of an enlarged posterior MA in patients with significant MR due to degenerative disease, while there was no evidence that the intertrigonal distance is abnormal in these patients, leading to the conclusion that posterior annular reduction with a flexible device at the time of MV repair is important, and that altering the anterior intertrigonal portion of the annulus is unnecessary (102). Changes in MA dynamics and long-term effects induced by annuloplasty in patients with organic prolapse undergoing MV repair were also investigated by 3D TTE (46). The authors showed that incomplete flexible band and complete semi-rigid rings induced similar dynamic MA changes and concluded that the main factor affecting annular function after annuloplasty is the undersizing of the MA induced by the ring, which restricts the MA geometry and limits the natural annular motion (46). In the same year, Maffessanti and colleagues evaluated the MV apparatus in 55 patients immediately before and after surgery, and in 18 controls (12). They used a dedicated commercial software that allows a quantitative assessment of the MV by 3D TEE data sets. MV repair and annuloplasty led to a significant undersizing of leaflets, and MA areas, diameters and height. Annular differences between BD and FED were reduced but still present after surgery (12). Several other studies have investigated the effect of various devices on annular shape and dynamics, with conflicting results (47, 52, 103–106). More importantly, in terms of clinical outcome, no clear difference between various solutions have been shown (107).

**Figure 6:**
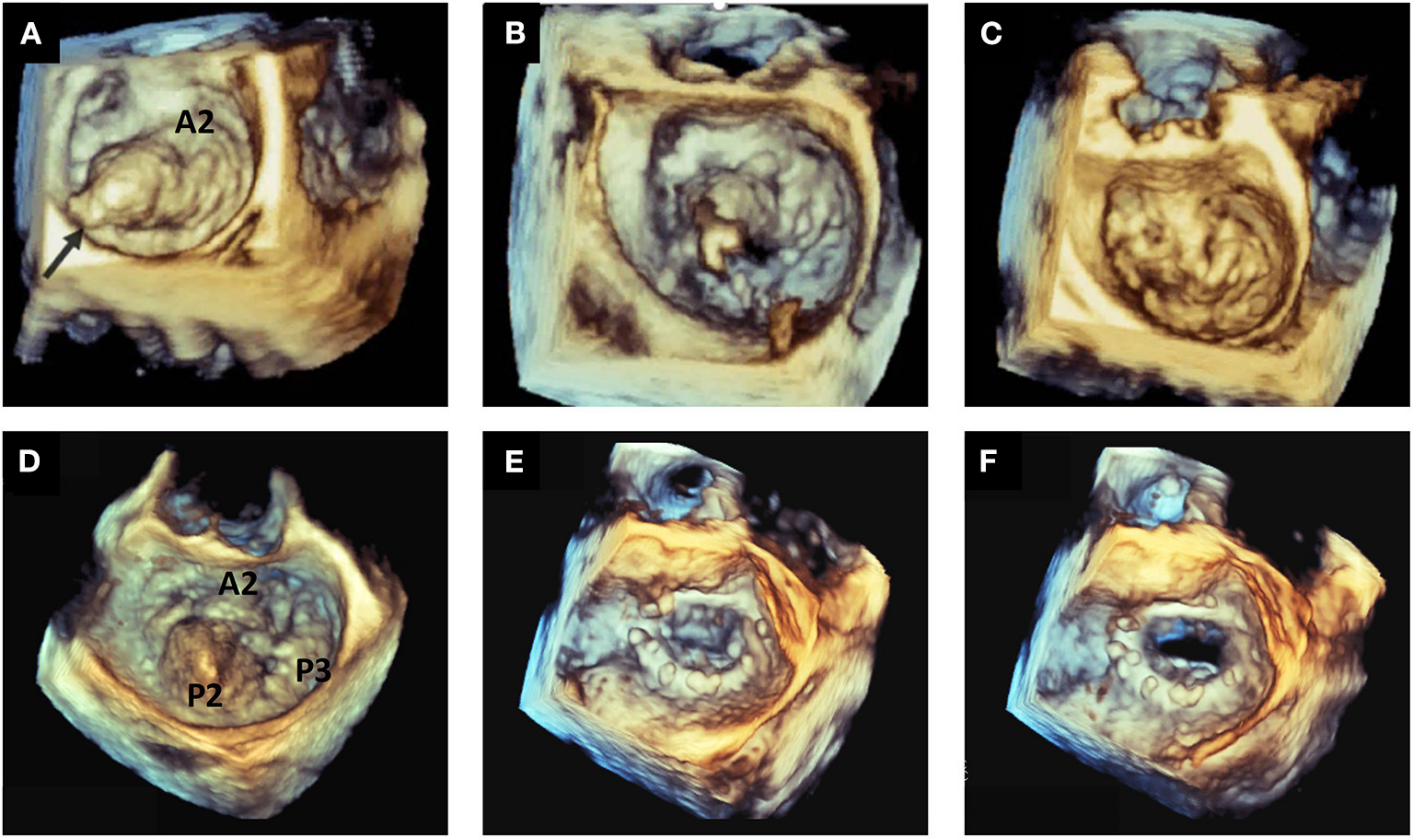
Examples of surgical mitral valve repair. Top panels show an example of A2 flail with single chordal rupture [arrow, **(A)**] corrected with the Neochord artificial chordae delivery system **(B)** obtaining the restoration of normal valve anatomy and competence **(C)**. Bottom panels show an example of multi-scallop prolapse involving A2–P2–P3 with a profound cleft between P2 and P3 **(D)**. The surgical repair was performed with a complete ring device, accurately visualized from the classical atrial perspective in systole **(E)** and diastole **(F)**.

In the setting of surgical MV repair, the contribution of 3DE is clear not only in the accurate diagnosis of MV pathology, but also in improving the communication and training between experienced and less trained operators, with optimal and comparable accuracy in the detection of MV lesions between echocardiographers and surgeons (108) (Figure 6).

### 4.3. Post-operative assessment of outcomes

3DE plays an important role also in the assessment of surgical outcomes. Kronzon et al. suggested that RT 3D TEE allowed the identification of the type of ring or prosthesis used, the description of the site, size, and shape of any dehisced segment, and the clear definition of the origin of residual MR. The additional information obtained by 3D TEE may be helpful in planning an appropriate subsequent intervention strategy (109).

## 5. Percutaneous treatment of mitral valve prolapse

Despite surgical correction is the treatment of choice for MR associated to MVP, it is often not feasible in high-risk patients. The EuroHeart survey reported that approximately half of the symptomatic patients with severe MR of functional and degenerative origin are denied surgery, and the likelihood of surgery denial increases with LV dysfunction, age and presence of comorbidities (83).

Percutaneous approaches to treat MR can be mainly categorized into leaflet repair, annuloplasty, chordal implantation, and transcatheter MV implantation (110). In Europe, several devices have received the Conformitee Europeene (CE) marking to treat primary MR, including the MitraClip (Abbott Laboratories, Abbott Park, IL), PASCAL (Edwards Lifesciences, Irvine, CA), NeoChord DS1000 (NeoChord, Inc, St. Louis Park, MN) and, most recently, Tendyne (Abbott Vascular, Santa Clara, CA). In the United States, the Edwards SAPIEN 3 transcatheter heart valve (Edwards Lifesciences) has received United States Food and Drug Administration (FDA) approval for MV replacement. Devices addressing MA dilatation, as Mitralign (Edwards Lifesciences), Carillon (Cardiac Dimensions; Kirkland, WA), Cardioband (Edwards Lifesciences), are currently tested for secondary MR. Among the percutaneous MV repair technologies, transcatheter edge-to-edge repair (TEER) with the MitraClip system or the PASCAL system has undergone the most extensive human investigation, has the most research data and the clearest evidence of therapeutic efficacy for all causes of MR.

### 5.1. Transcatheter edge-to-edge MV repair

TEER mimics the surgical MV repair procedure introduced by Alfieri, who treated a patient with an anterior prolapse by placing a pledgeted stitch to approximate the middle portions of the MV leaflets, creating a double orifice MV and successfully reducing MR (111). After insertion *via* the femoral vein and transseptal access, the device is positioned over the maximum MR area on the atrial side of the MV and aligned perpendicular to the line of coaptation. After advancing into the LV, corresponding segments of the anterior and posterior mitral leaflets are grasped on retraction.

The EVEREST trials (I and II) and pivotal studies established the feasibility and safety of the MitraClip procedure and ascertained a hemodynamic improvement and symptom alleviation in the large majority of patients, despite a less effective MR reduction than conventional surgery, and thus despite a higher rate of reintervention and/or surgical operations (112–115).

Echocardiography is the essential imaging modality for TEER, specifically for patient selection, guidance of the procedure, evaluation of the result after implantation, and assessment at follow-up of residual MR severity, and LV size and function (116, 117) (Table 3). 3D echocardiography may provide new additional and useful data in each of these scopes.

**Table 3:** Role of echocardiography for planning and guiding transcatheter edge-to-edge leaflet repair in mitral valve prolapse.

**Pre-procedural evaluation**
3D assessment of MV anatomy and identification of leaflet abnormalities
Measurement of 3D VCA, location of the regurgitant orifice and identification of multiple MR jets
**Favorable echocardiographic features**
A2/P2 prolapse
Flail gap <10 mm and flail width <15 mm
Non-tethered leaflets with leaflet length ≥10 mm
MV area >4 cm^2^ and baseline MV mean gradient <3 mmHg
Single central jet or jet has a dominant central location
Transseptal crossing height to MA plane >4 cm
**Challenging echocardiographic features**
Barlow's disease
Commissural or anterior leaflet prolapse
Multisegmented prolapse/flail
Significant cleft; leaflet or chordal calcification within the grasping zone;
leaflet perforation
Severe MA calcification, with <5 mm of leaflet available for grasp
Posterior mitral leaflet length <7 mm
MV area <4 cm^2^ and baseline MV mean gradient 4–5 mmHg
Small LA size
**Intraprocedural guidance**
Safe and optimal site of transseptal puncture
Introduction of the steerable guide catheter into the LA and advancement of the clip delivery system
Safe steering of the clip delivery system and its alignment perpendicular to the MV coaptation plane
Adequate grasping of the leaflets
Assessment of MR reduction (3D VCA), device release and exclusion of significant mitral stenosis (3D TEE MV area)

3D, three-dimensional; LA, left atrium; MA, mitral annulus; MR, mitral regurgitation; MV, mitral valve; VCA, vena contracta area.

**Figure 7:**
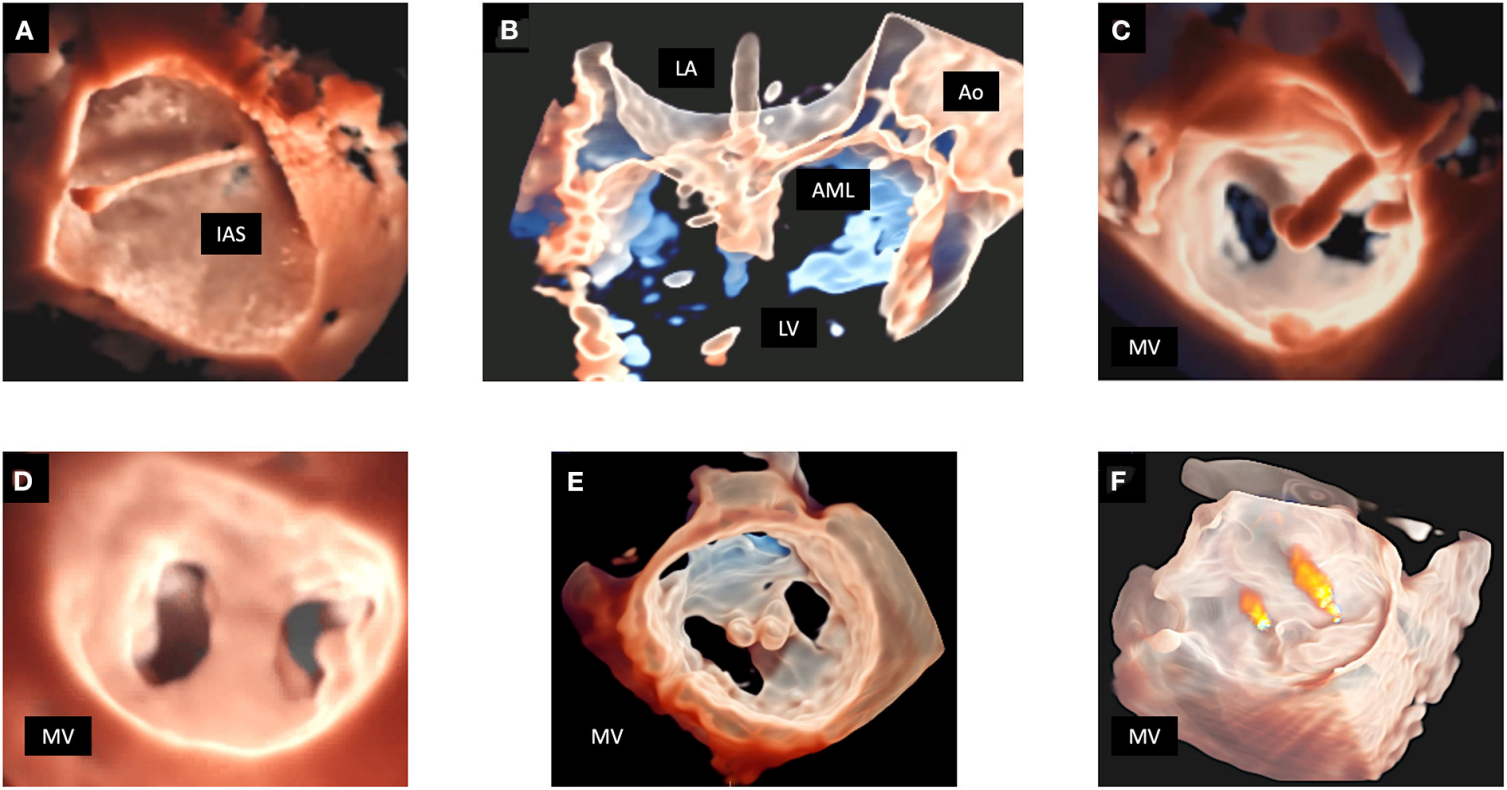
MitraClip procedure monitoring in a patient with MV prolapse. In **(A)** TI allows a detailed depiction of the trajectory across the interatrial septum into the left atrium. In **(B)** the “transparency” effect clearly shows the capture of the MV leaflets by the clip. In **(C)** TI rendering enhances the visualization the clip before deciding for its release. The lower panels show the result of the procedure. 3D MV reconstruction is displayed by TI rendering from the LA perspective **(D)**, and from the LV perspective with the application of the “transparency” effect, clearly showing 2 clips implanted in the correct position **(E)**. In **(F)** the residual regurgitant jets are displayed in a 3D reconstruction of the MV with the “transparency” effect and superimposed color Doppler. 3D, three-dimensional; AML, anterior mitral leaflet; Ao, aorta; IAS, interatrial septum; LA, left atrium; LV, left ventricle; MV, mitral valve; TEE, transesophageal echocardiography; TI, transillumination. TTE, transthoracic echocardiography.

Patients from the initial experience had to fulfill strict echocardiographic criteria to be considered suitable for TEER (Table 3). Nowadays, the large accumulated experience in parallel with continuous technical advancements broadened the indications of the procedure. Therefore, selection of candidates and planning of the procedure requires a very detailed analysis by 2D and 3D TEE (Table 3). Indeed, it is well-known that 3D TEE provides a detailed assessment of the MV anatomy and function, and all components of the mitral apparatus and adjacent structures. 3D TEE is superior in the detection of clefts, gaps, and perforations which are frequently missed by 2D TEE evaluation (20). Although 2D TEE can be used as the sole method for guidance of TEER, 3D TEE provides valuable additional information and advantages in almost every step of the procedure: the more precise anatomic information, the fine details of the devices and the precise relationship of catheters and devices with surrounding anatomic structures may enhance the confidence of imaging interpretation and eventually improve the efficiency of the procedure (1, 118, 119) (Table 3). Biner et al. demonstrated that the combined use of 2D and 3D TEE was associated with a shorter time to first clip deployment and with a general reduction in the total procedure time when compared to traditional 2D TEE guidance alone (120). Moreover, the use of 3D color Doppler allows a better identification of the site of origin and the degree of any residual intraprocedural MR jet, which is crucial when a second device has to be implanted. While in the past obtaining 3D color Doppler images required multi-beats image acquisition with possible stich artifact, recently available RT single-beat 3D color image acquisition allows instant discussion and communication between echocardiographers and interventionalists directly watching images. Another crucial step during the percutaneous edge-to-edge repair of the MV is the assessment of device attachment to the MV leaflets, which may be challenging using 2D TEE (Table 3). In the EVEREST I trial, single leaflet clip detachment occurred in 9% of the patients, demonstrating the need for new techniques that allow a more reliable assessment of clip attachment (112). Braun et al. demonstrated that 3D TEE can quantify exactly the portion of MV leaflets fixed into the clip by measuring the distance between the lowermost part of the leaflet and the top edge of the clip (121). They found that the frequency of clip complications (partial clip detachment or displacement) was higher in patients imaged by 2D TEE compared to patients imaged by 3D TEE (121). Recently, new 3D rendering tools (TI and “transparency” effect) furtherly improve the visualization and delineation of MV anatomy and pathology, and improve localization of regurgitant jets compared with standard 3D rendering (32, 34). 3D TEE imaging during procedures might be improved by TI and the “transparency” effect providing a better evaluation of leaflet grasping and of residual MR jets (35) (Figure 7).

Echocardiography plays an important role also in grading post-procedural MR, that is a challenging and an unsettled issue. The presence of residual MR is easy to detect with color Doppler but, to date, no single echocardiographic method has been recommended for post-procedural MR quantification, with the evaluation currently relying on a complex, multiparametric appraisal (122). The direct measurement of 3D VCA shows potential for MR quantification, although currently no reference data are available (122, 123) (Table 3).

TEER alters the MV morphology acting not only on the leaflets, but also on the annular geometry, and a correlation between baseline geometry and cardiac remodeling has been demonstrated. Specifically, a pre-procedural AP diameter <4.44 cm at 3D TEE seems to be a potential predictor of mid-term optimal result, intended as a reduction in MR grade ≥ 2 and favorable LV remodeling at 6-months follow-up (50). Kim et al. used 3D TEE to measure TEER-induced changes in annular geometry. They confirmed that suboptimal response (residual MR grade >mild) was associated with larger pre-procedural MA area on 3D TEE, and found that the magnitude of reductions in MA circumference by intra-procedural 3D TEE was greater among patients with, compared to those without, suboptimal response on follow-up TTE (124).

All these results support the importance of 3D imaging for pre-procedural patient selection, intraprocedural guidance and prediction of procedural effectiveness.

## 6. Future perspectives

Emerging 3D imaging technologies are furtherly enhancing our understanding of the anatomy of cardiac structures and the spatial relationship among them. These techniques increase diagnostic accuracy, allow to produce 3D printing of patient-specific cardiac structures, allow simulation and planning of surgical and percutaneous procedures, and add significant information during interventions. Despite the incremental value of these imaging modalities has not been established yet, several studies describe their initial application to MV disease.

In the last decade, 3D TEE and fluoroscopy fusion software has been developed, providing a live 3D model showing a heart beating over fluoroscopy during structural MV interventions. Based on artificial intelligence algorithms, the model highlights anatomical landmarks, and by using 3D color Doppler TEE imaging, the information about MV flow and regurgitant jets is added to the fused 3D model. Fusion of TEE with fluoroscopy provides procedural operators with real-time guidance, ensuring a precise and safe manipulation of catheters and implantable devices into heart chambers during TEER, transcatheter annuloplasty and transapical chordal replacement (125–127). Other novel techniques applying advanced cardiac multimodality imaging are fusion of pre-procedural computed tomography (CT) with peri-procedural live fluoroscopy and 3D TEE, respectively. This is particularly useful during transcatheter MV replacement interventions, where CT landmarks constitute the road map for the procedure (127, 128). Development of vendor-independent software fusing RT 3D TEE, CT and fluoroscopy may further improve MV procedural guidance and success (127).

A new additional tool is virtual reality derived from 3DE data sets by using dedicated software. With the aid of a head-mounted display, virtual reality allows the operator to immerse in a virtual 3D space, covering the whole field of view. Data obtained from 3D TEE can be projected as a real-time 3D hologram representing cardiac structures that can be manipulated by the operator using handheld controllers (129). Similarly, augmented reality illustrates virtual elements in a real-world environment integrating specific imaging modalities into reality. Although these tools may improve visualization of MVP and be beneficial for pre-procedural individualized planning of MV interventions, their usage still needs to be validated in routine clinical practice.

Finally, the emergence of artificial intelligence methods might facilitate the interpretation of echocardiographic images and revolutionize MVP diagnosis. A preliminary study was published demonstrating that artificial intelligence provides an automated, accurate and rapid localization of MVP compared to the manual approach by less experienced operators (130).

## 7. Conclusion

Echocardiography is an essential diagnostic tool in the evaluation of patients with MVP. In this context, 3DE provides several advantages compared to 2D echocardiography. 3DE contributes to the differentiation between leaflet prolapse and billowing, and to the identification of the underlying etiopathogenetic process. Also, it increases the accuracy in the recognition of the site and extent of MV lesions and improves the understanding of MA dynamics and MR pathophysiology, especially by RT 3D TEE. The accuracy in the assessment of MR severity has significantly been improved by the advent of 3D. However, 3DE remains underused for this purpose in Routine clinical practice since direct measurement of the regurgitant jet 3D area is time-consuming and requires some experience. During surgical procedures, pre- and intra-operative 3D “en face” display of the MV obtained by TEE can give new insights into the mechanisms of MR and help the heart team members communicate with each other. Finally, 3D TEE is crucial for pre- and intra-procedural assessment of MV anatomy and MR mechanism, as well as for guidance of the procedure itself.

## Author contributions

VM and MP outlined the manuscript. VM and PG drafted and contributed to the writing of the manuscript. All authors approved the final version of the manuscript.
